# Effects of American Ginseng Cultivation on Bacterial Community Structure and Responses of Soil Nutrients in Different Ecological Niches

**DOI:** 10.4014/jmb.2202.02003

**Published:** 2022-02-23

**Authors:** Fan Chang, Fengan Jia, Rui Lv, Min Guan, Qingan Jia, Yan Sun, Zhi Li

**Affiliations:** 1 College of Life Science, Shaanxi Normal University, Xi’an 710062, P.R. China; 2 Shaanxi Institute of Microbiology, Xi’an 710043, P.R. China; 3 Shaanxi Agricultural Machinery Research Institute, Xianyang 712000, P.R. China; 4 Institute of Medical Research, Northwestern Polytechnical University, Xi'an 710072, P.R. China

**Keywords:** American ginseng, cultivation mode, bacterial community, chemical properties

## Abstract

American ginseng (*Panax quinquefolium* L.) is a perennial herbaceous plant widely cultivated in China, Korea, the United States, and Japan due to its multifunctional properties. In northwest China, transplanting after 2-3 years has become the main mode of artificial cultivation of American ginseng. However, the effects of the cultivation process on the chemical properties of the soil and bacterial community remain poorly understood. Hence, in the present study, high-throughput sequencing and soil chemical analyses were applied to investigate the differences between bacterial communities and nutrition driver factors in the soil during the cultivation of American ginseng. The responses of soil nutrition in different ecological niches were also determined with the results indicating that the cultivation of American ginseng significantly increased the soluble nutrients in the soil. Moreover, the bacterial diversity fluctuated with cultivation years, and 4-year-old ginseng roots had low bacterial diversity and evenness. In the first two years of cultivation, the bacterial community was more sensitive to soil nutrition compared to the last two years. Proteobacteria, Actinobacteria, Gemmatimonadetes, Acidobacteria, Firmicutes, and Bacteroidetes dominated the bacterial community regardless of the cultivation year and ecological niche. With the increase of cultivation years, the assembly of bacterial communities changed from stochastic to deterministic processes. The high abundance of *Sphingobium*, *Novosphingobium*, and *Rhizorhabdus* enriched in 4-years-old ginseng roots was mainly associated with variations in the available potassium (AK), total phosphorus (TP), total potassium (TK), and organic matter (OM).

## Introduction

American ginseng (*Panax quinquefolius* L.) is one of the most well-known medicinal herbs and is widely cultivated worldwide [1]. Due to its important pharmacological properties and applications, including enhancing the central nervous system, protecting the cardiovascular system, improving immunity, stimulating blood flow, as well as assisting the treatment of diabetes, American ginseng has been used as a “cool” medicinal herb based on the traditional Chinese medicine (TCM) theory for almost 300 years [2, 3]. Originally from southeast Canada and the northern USA, American ginseng has been cultivated in China since the 1980s [4]. In northwestern China, a “two-year land-changing planting” transplanting mode is generally adopted. In this case, the ginseng is transplanted to new soil after two years of growth. The main reason for this mode of cultivation is that American ginseng cannot be consecutively cultivated on the same plot of land year after year due to its high soil quality requirements [5]. However, after transplanting, the continuous cropping obstacle occurs in the two fields cultivated with American ginseng. Continuous cropping obstacles, also known as replanting disorders [6], are a pressing and hard-to-control problem in herb cultivation worldwide. This problem has also seriously restricted the sustainable development of American ginseng.

Many factors, both abiotic and biotic, can lead to continuous cropping obstacles in the cultivation of Chinese medicinal herbs. The physicochemical characteristics of the soil are the main abiotic factors that present continuous cropping obstacles [7-10], including soil nutrients and allelopathic autotoxicity in plants. For example, Zhang *et al*. [11] reported that the ecological structure of microbes and the balance of soil nutrients changed after American ginseng cultivation. These changes affected the soil pH and decreased the microbial diversity, which also led to the inhibition of American ginseng growth and increased the incidence of diseases. Biotic factors often included bacterial and fungal communities, protozoa, and insect pests [12, 13]. Most biological diseases are caused by soil-borne pathogenic fungi but other biological factors can also contribute to disease [14, 15]. Previous studies have also found that some beneficial bacteria can participate in soil structure improvements. For example, bacteria with high antagonistic potential can suppress soil-borne pathogens [5].

Furthermore, increasing evidence indicates that microorganisms in the soil and rhizosphere play a vital role in sustaining agro-ecosystems and mediating many biological processes, including nutrient cycling and bioremediation [6]. Additionally, in a particular type of habitat, the microbiota is often mentioned [16, 17]. With the development of high-throughput DNA sequencing technologies, studies regarding the community diversity and structure of soil microorganisms have increased and helped in understanding the relationships and interactions between microbiome and soil characteristics during Araliaceae cultivation [12, 13, 18]. On the other hand, there are few studies on the bacterial diversity and structure of soil and root microbiome during American ginseng cultivation in the transplanting mode, especially regarding the bacterial community of replanting or abandoned soil. Therefore, in the present study, we analyzed the soil biochemical characteristics and bacterial communities of American ginseng during the cultivation period. We combined high-throughput sequencing with bioinformatics to analyze the richness and diversity of the 16S rRNA gene V3–V4 region of soil bacteria after DNA extraction, PCR amplification, and database construction. Overall, we provided a theoretical basis for the understanding of changes in American ginseng microbial communities and soil chemical properties during cultivation years.

## Materials and Methods

### Experimental Site and Soil Collection

The experiment was conducted in Liuba (106°52′N, 33°40′E), Shaanxi Province, which is the only ginseng planting area in northwest China. American ginseng was cultivated on new reclaimed farmland having no history of agricultural utilization. Organic fertilizer (4.0 kg/m^2^) was applied as the base fertilizer before the cultivation of American ginseng and added (4.0 kg/m^2^) once a year in March. The soil moisture content was adjusted to 40–50%. The cultivation followed the standard operating procedures of Good Agricultural Practice (GAP) [19]. Depending on the management and years of American ginseng cultivation, we selected the following soil sampling groups: uncultivated soils (UCS); cultivation in virgin land for one year (VY1); cultivation in virgin land for two years (VY2); 2-year-old American ginseng transplanted to a new virgin field for the third year (VY3); 2-year-old American ginseng transplanted to a new virgin field for the fourth year (VY4); American ginseng post-harvest soil abandoned for four years (PH); and 4-year-old American ginseng cultivation on continuous cropping land after crop rotation for 10 years (CRY4). Samples were collected from the planting field and ginseng root rhizosphere soils of American ginseng in growth stages. During cultivation, field plots were arranged in a randomized block design with three replicate plots (1.5 × 10 m). For planting field soil (S groups), five soil samples per planting plot were collected based on the five-spot-sampling method, and each sample was thoroughly mixed to form a composite. Then, they were homogenized using a 2-mm sieve and separated in triplicate into sterile bags for detection of bacterial diversity, soil chemical properties, and a backup. For ginseng root rhizosphere soil (R groups), 5–7 roots (20~30 ginseng roots for 1- and 2-year groups) were randomly selected, ultrasonically vibrated in sterile water, and centrifuged to collect soil samples [20, 21]. In total, 21 soil and 15 rhizosphere samples were collected. All soil samples were transported to the laboratory in a cold chain and aseptic bags. They were treated three times with liquid nitrogen and stored at -80°C as soon as possible until further analyses.

### DNA Extraction, PCR Amplification, and Sequencing

Genomic DNA was extracted from 0.5 g of soil collected from four locations using the Fast DNA Spin Kit for Soil (MP Biomedicals, USA) with a final elution volume of 100 μl, following the manufacturer’s instructions. Then, the isolated microbial DNA was used as a template for sequencing. The yield and quality were determined using an Agilent 2100 Bioanalyzer (Agilent Technologies, USA).

For prokaryotic 16S rDNA, the V3 and V4 hypervariable regions were selected for amplicon generation and subsequent taxonomic analysis. The V3 and V4 regions were amplified using the following primers: 341F (forward primer 5'-CCTACGGGNGGCWGCAG-3') and 805R (reverse primer 5'-GACTACHVGGGTATCTAATCC-3'). The PCR conditions were: the reaction mixture (70 μl) contained 0.7 μmol of each primer, 200 μmol of dNTPs, 10× Ex Taq reaction buffer, and one unit of Ex Taq DNA polymerase (Takara, Japan); 31 cycles of 94°C for 30 s, 52°C for 30 s, and 72°C for 45 s in an Applied Biosystems thermal cycler (GeneAmp PCR system 2700). Next-generation sequencing (NGS) preparation, Illumina HiSeq NGS library preparations, and Illumina HiSeq sequencing were conducted at Novogene Inc. (China). DNA samples were quantified using a Qubit 2.0 fluorometer (Invitrogen, USA). Then, 30-50 ng DNA was used to generate amplicons using the NEBNext UltraTM DNA Library Prep Kit for Illumina^®^ (New England Biolabs, USA) according to the manufacturer’s protocol.

### Sequence Processing and Taxonomic Affiliation

The Usearch10 [22] and Vsearch 2.8.1 [23] data analysis pipelines were used to evaluate the 16S rRNA data. First, forward and reverse reads were joined, assigned to samples based on barcodes, and truncated by the removal of the barcode and primer sequences. Quality filtering was performed on joined sequences, and the sequences that did not fulfill the following criteria were discarded: no ambiguous bases and expected errors per base rate > 0.01. Then, the sequences were dereplicated, and singletons (minuniquesize < 8) were removed. Next, the sequences were clustered into amplicon sequence variants (ASVs) using the exact sequence variants algorithm [24, 25](Unoise3), and chimeric sequences were simultaneously removed. The effective sequences were used in the final analyses. Sequences were grouped using the clustering program VSEARCH 2.8.1 against the Ribosomal Database Program (RDP, http://rdp.cme.msu.edu/) and preclustered at 97% sequence identity. The RDP classifier [26] was used to assign taxonomic categories to all ASVs at a confidence threshold of 0.8.

### Detection of Soil Physicochemical Properties

First, samples were air-dried in a soil drying room and used to estimate organic matter (OM), total nitrogen (TN), amino nitrogen (AN), total phosphorus (TP), Olsen-P (OP), total potassium (TK), and available potassium (AK) (Qu *et al*. 2019). Organic matter was measured by sulfuric acid-potassium dichromate wet oxidation, followed by titration with ferrous sulfate according to Walkley-Black [27]. The total N was determined by the Kjeldahl method [28] and soil available nitrogen (AN) was determined by diffusion methods [29]. The TP in the soil was measured by the Mo-Sb anti-spectrophotometric method [30] and the OP by the Olsen method [31]. The TK and AK in the soil were determined using a flame photometer after ammonium acetate extraction [32].

### Data Analyses

All statistical analyses were performed using MicrobiomeAnalyst (https://www.microbiomeanalyst.ca/) [33] and R software version 4.1.2. For taxonomic analysis, the alpha-diversity was evaluated using the Chao1 index with the “vegan” package (version 2.5-6, https://CRAN.R-project.org/package=vegan) [34]. Phylogenetic tree analysis of soil was conducted using *cluster_agg* from Usearch10. The alpha-diversity indices were compared by one-way analysis of variance (ANOVA) and Tukey's multiple comparisons test.

The overall soil treatment effect on bacterial communities was examined using principal coordinates analysis (PCoA) combined with multivariate PERMANOVA of Bray-Curtis distances based on ASVs level and computed with the “vegan” R package. Further, to explore the structure of bacterial community assembly processes by deterministic or stochastic processes, the β-nearest taxon index (βNTI) [35] was calculated with the “picante” package (version 1.8.2). The bacterial community assembly processes can be indicated by the fraction of communities with |βNTI|>2 and |βNTI|<2, respectively. The linear discriminant analysis effect size (LEfSe) method was used [36] to compare microbial compositions of the soil amendments. The correlation between the phylum and environmental variables was assessed by redundancy analysis (RDA) using the “vegan” R package. To evaluate their associations with the community structures, environmental factors were transformed and normalized for use in the Mantel test [37]. For soil chemical property values, group comparisons were performed using the Wilcoxon/Kruskal-Wallis test.

## Results

### Soil Chemical Properties

Compared with UCS, the cultivation of American ginseng significantly increased the soil AN, AK, OP, and TP levels. These levels were highest in the fourth year of cultivation, indicating that soil nutrients might have exceeded the requirements for American ginseng growth. In the first and second years of cultivation, the soil nutrients did not significantly change. The fastest-growing soil nutrient was AK (Table 1).

### Analysis of Sequencing Data and Estimation of Bacterial Diversity

After filtering and chimera removal, 36 samples yielded a total of 1,972,074 high-quality sequences, with an average of 54,779 sequences for each sample. High-quality sequences were clustered into 13,856 microbial ASVs using Unoise3 sequence identity.

**Fig. 1:**
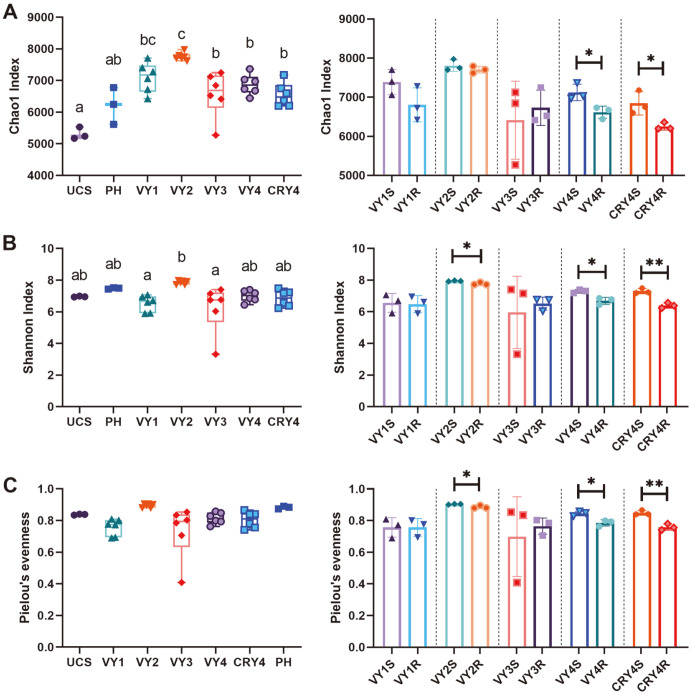
Effects of cultivation years and niche on bacterial richness and evenness of American ginseng. (**A**) Chao1, (**B**) Shannon, and (**C**) Pielou’s evenness indices. Tukey's multiple comparisons test was performed with various groups to compare the differences. Values with the same letters did not significantly differ. The significance is represented by * (*p* < 0.05) and ** (*p* < 0.01).

To determine the changes of bacterial community between different years of cultivation, the richness, diversity, and evenness of bacterial ASVs were evaluated using the Chao1, Shannon, and Pielou’s evenness indices. Different indices had diverse effects on the richness and diversity of soil bacterial community. According to one-way ANOVA, the growing years significantly affected the Chao1 richness index. With cultivation years, this index showed a trend of slow decline with a rapid increase (Fig. 1A). For the UCS group, this index was significantly lower compared to all cultivation years, suggesting that the cultivation process significantly affected bacterial richness. The Shannon index followed the same trend as the Chao1, with the highest value in the second year (Fig. 1B). No significant differences were detected in the Pielou’s evenness in all years of cultivation (Fig. 1C).

In addition to the planting years, different ecological niches also affected bacterial diversity indexes. After American ginseng transplanting, the soil Chao1, Shannon, and Pielou’s evenness indices were significantly higher compared to ginseng roots, similar to both virgin (VY) and crop rotation (CRY) fields. We also observed significant differences in Shannon and Pielou’s evenness indices between soil and ginseng roots in the second year of American ginseng cultivation (Fig. 1).

### Variability of Bacterial Community Structures during the Cultivation Process

**Fig. 2:**
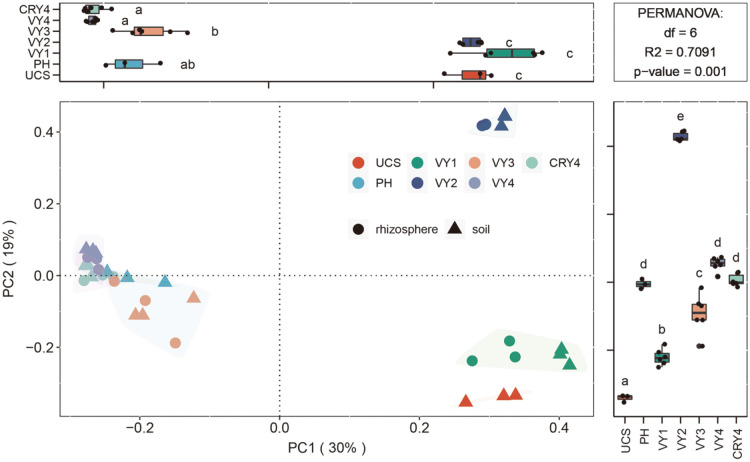
Two-dimensional PCoA analysis ranking of four years of the cultivation process. Each colored and shaped dot represents a sample.

The PCoA was performed to determine differences in bacterial community structures based on Bray–Curtis distances, in which the horizontal (PC1 axis) and vertical (PC2 axis) coordinates were the principal components contributing to the differences in the bacterial community composition among all samples (Fig. 2).

Collectively, PCoA contributed 49% variation to the bacterial community composition of all samples (PC1, 30% and PC2, 19%) (Fig. 2), indicating that a difference in American ginseng cultivation years was the major influencing factor (*p*-value = 0.001, permutational multivariate analysis of variance (PERMANOVA) by Adonis). A strong difference was observed in microbial communities in UCS, VY1 and VY2 groups, and this was captured by their clustered distributions. In addition, it was observed that the effect of niche on bacterial community structure may play a major role in the late cultivation period. The short distance in VY3, VY4, and CRY4 indicated the disturbance of niche on bacterial diversity mainly occurred in the late cultivation period (Fig. 2).

**Fig. 3:**
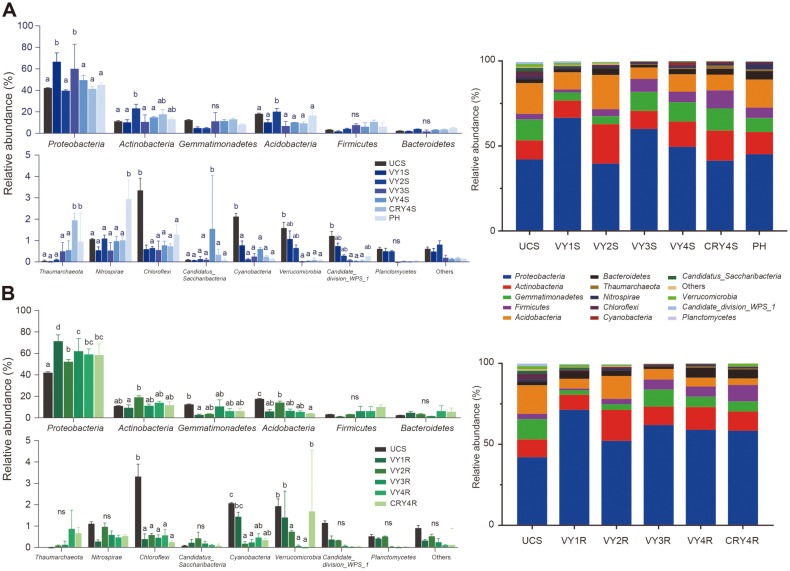
ASV-based community compositions and differences in the relative abundance of bacterial phyla over American ginseng cultivation years. Relative abundances and differences of bacterial phyla in (**A**) soils, and (**B**) ginsengs. Values with the same letters did not significantly differ.

Based on the RDP database, the bacterial ASVs were classified and clustered at the phylum level. The relative abundance of the top 15 bacterial phyla is shown by bar plots in Fig. 3. The dominant bacterial phyla (relative abundance > 1%) in the community were Proteobacteria, Actinobacteria, Gemmatimonadetes, Acidobacteria, Firmicutes, and Bacteroidetes. Their relative abundance showed a similar trend with the cultivation years, regardless of the ecological niche. Compared to UCS, the relative abundance of Proteobacteria significantly increased in 1- and 3-year-old American ginseng cultivations. For Actinobacteria and Acidobacteria, the relative abundance significantly increased after two years of cultivation compared to UCS. Although the changes were not significant, Gemmatimonadetes, Firmicutes, and Bacteroidetes showed a similar trend with the cultivation years. On the other hand, some relative abundances among the top 15 phyla showed different trends with diverse ecological niches (ginseng roots or bulk soils) and planting years. Compared to other groups, the relative abundance of Thaumarchaeota significantly increased in CRY4S but not in CRY4R.

### Assemblage Processes of the Bacterial Community with Cultivation Years in Ginseng and Bulk Soil Niches

Both deterministic and stochastic processes are responsible for structuring microbial communities [38]. The βNTI scores for the ASV-derived communities increased with cultivation years, indicating the dominant role of deterministic processes. Most βNTI scores in the four years of cultivation were > 2, which showed that deterministic processes dominated the bacterial community dynamics during the four years of American ginseng cultivation (Fig. 3A). Moreover, deterministic processes with cultivation years dominated the bacterial community dynamics in both ginseng roots and soil niches, regardless of cultivation in virgin or rotation lands (Fig. 3B). Additionally, all βNTI scores in 4-year-old soils were > 2, suggesting that the deterministic process in the 4^th^ year made a major contribution to soil bacterial communities. The community of ginseng roots also developed to a deterministic process at the same time (Fig. 3B).

### Bacterial Community of Different Ecological Niches of American Ginseng at the Late Cultivation Stage

In the 4^th^ year of American ginseng cultivation, the bacterial community and structure of bulk soils and ginseng roots changed greatly. Hence, we explored the bacterial community structure and differences in relative abundance at the genus level in this period. The PCoA analysis revealed that, unlike previous results, ginseng roots and soil were closer to each other in the 4^th^ year of cultivation, with a significant separation on the PC1 axis (Fig. 5A). The clustering analysis is displayed in the form of a hierarchical tree and revealed that, in the 4^th^ year of cultivation, the bacterial community structures of ginseng roots and bulk soils were clustered separately (Fig. 5B). Further, we used the LEfSe analysis to identify significant differential bacterial genera of ginseng roots and bulk soils in the 4^th^ year of cultivation (Fig. 5C). We found that more discriminative genera appeared in the bulk soil groups (21), while only seven were detected in ginseng roots.

Furthermore, we compared the relative abundance of the first seven differential genera in soils and ginseng roots (Fig. 5D). Among the discriminative genera in bulk soil groups, the mean relative abundance of all genera was higher than 1%, except for *Rhizomicrobium* and *Stella*. The genera *Azospirillum*, *Rhizomicrobium*, and *Stella* belong to the phylum Firmicutes, and *Nocardioides* and *Ilumatobacter* to the Actinobacteria. The highest relative abundance of Gp16 belongs to the phylum Acidobacteria. Additionally, *Nitrospira*, belonging to *Nitrospira*e, was also found in soil groups. These discriminative bacterial genera belonging to different phyla indicated the complexity of the microbial community structure in the 4^th^ year of American ginseng cultivation. Meanwhile, in the ginseng root group, the differential bacterial genera were mainly concentrated in Bacteroidetes (*Pedobacter* and *Flavobacterium*), Proteobacteria (*Sphingobium*, *Novosphingobium*, *Rhizobium*, and *Rhizorhabdus*), and the *Microbacterium* (Actinobacteria). Unlike the complexity of soil groups, the discriminative bacterial genera of ginseng root groups were clustered in a few phyla, especially Proteobacteria. The average relative abundance of *Novosphingobium* and *Rhizobium* was over 4% each, and for *Sphingobium* was over 9%.

### Correlations between Bacterial Community and Edaphic Chemical Properties with American Ginseng Cultivation

The relationships between different niches, bacterial communities (at the genus level), cultivation years, and edaphic physicochemical properties were explored using Redundancy analyses (RDA). For the ginseng niche, 73.23% of the overall bacterial community composition variability was explained by the first two principal components (Fig. 6A). The effects of AK were greater than other physicochemical factors and positively correlated with the genera of the 4-year-old cultivation group (VY4 and CRY4), including ginseng discriminative bacterial genera. The Mantel test showed that AK and TP were strongly associated (Mantel’s R > 0.3, *p* < 0.01) with ginseng bacterial community structures at the genus level. The OM, TP, TK, and TN also had a significant effect on the community structures of ginseng discriminative genera (LEfSe ginseng genus). Finally, we observed that AK had a significant effect on the structures of other undifferentiated genera communities (Fig. 6A).

For the soil niche, we observed the effects of bacterial community complexity on the physicochemical properties. The redundancy analysis showed that 53.84% of the overall bacterial community composition variability was explained by the first two principal components. A clear separation of coordinate positions with American ginseng cultivation years was also detected. However, no physicochemical factors were significantly associated with the soil bacterial community structure (Fig. 6B).

## Discussion

### Effects of the Cultivation Process on Soil Nutrients, Bacterial Diversity, and Bacterial Evenness

Soil nutrients are considered an important factor in improving the stress resistance and yield of American ginseng [39]. Additionally, the transplantation mode directly affects the changes of soil nutrients during its growth [40]. Similarly, in the present study, the VY4 and CRY4 groups (4^th^ year of American ginseng cultivation soil) were rich in AN, OP, and AK (Table 1). We hypothesized that the accumulation of these soil-available nutrients was due to higher inputs of chemical fertilizers and extensive application of organic fertilizers. Interestingly, we showed that, when the TK content was relatively stable, the AK content significantly increased with cultivation years, especially in the 4^th^ year. Our findings were consistent with previous research showing that the growth of American ginseng affected the composition of the soil [41].

Another interesting finding was that the transplantation mode had a significant impact on bacterial richness and diversity. The Chao1 and Shannon indices of bacteria significantly decreased after transplanting, which was also shown in the re-cultivation of American ginseng after field rotation (CRY4). Although it has been reported that cultivation years can lead to a decrease in bacterial diversity [6], we found that Chao1, Shannon, and Pielou’s indices of ginseng roots were significantly lower compared to the soil in the fourth year of cultivation (Fig. 2A). These results suggested that the 4-year-old ginseng root niche might affect the bacterial richness, diversity, and evenness. Although Zhao [42] found that the planting process reduced the soil bacteria evenness, we found that the Pielou’s evenness index remained stable throughout the American ginseng cultivation period. We also found that the bacterial diversity and evenness of ginseng roots decreased in the second and fourth years of cultivation. This niche can directionally select specific microbial communities and symbionts, and benefit ginseng plants [43], which would explain the decrease of ginseng root bacterial evenness.

### Effects of the Cultivation Process on Bacterial Community Structure

The bacterial community structure gradually stabilized with the cultivation years of American ginseng, consistent with previous reports [11]. In the first and second years of cultivation, the bacterial community structure fluctuated greatly with planting years, which could be observed from the relative distance between VY1 and VY2. After transplantation, the distance between the third and the fourth years reduced, indicating that the structure of the bacterial communities was more similar in these periods (Fig. 2). Some studies showed that the transplantation mode also had a significant impact on the bacterial community structure [40], consistent with our current results. The first and second years of cultivation were significantly separated along the PC1 axis from the third and fourth years.

Although the relative abundance and variation trend of different niches were similar at the phylum level (Fig. 3), we found that the ginseng roots seemed to be more enriched of *Sphingomonadaceae* at the family level (Fig. 7), especially in the 3^rd^ and 4^th^ years after transplantation. The relative abundance of *Sphingomonadaceae* and Gemmatimonadaceae in soil was similar to ginseng roots. Proteobacteria are the most abundant phylum in American ginseng soil, including many families [44, 45]. Interestingly, we found that *Pseudomonadaceae* and *Xanthomonadaceae*, which were previously associated with disease suppression [46], were the only abundant groups in the soils of American ginseng cultivation in the 1^st^ and 2^nd^ years (Fig. 7). Actinobacteria was the second most dominant phylum, which is reported to be involved in the degradation of organic matter in soils [47]. Similar to our findings, the primary bacteria in American ginseng soils were Proteobacteria and Actinobacteria [11, 45]. In our present study, the relative abundance of Proteobacteria and Actinobacteria exceeded 50% in all samples, presenting a potential role in the microbial homeostasis of American ginseng soils (Fig. 3). Moreover, we found that the relative abundance of Actinobacteria was relatively low at the family level, but this is a large category (Fig. 7). In phyla with low relative abundances, such as *Nitrospira*e, the trend of changes was different for diverse ecological niches. Previously, a significant enrichment of *Nitrospira*e in abandoned soil (PH group) was reported to be related to rusty root disease [48].

### Assemblage Processes and Responses of the American Ginseng Bacterial Community to Different Ecological Niches during Cultivation

**Fig. 4:**
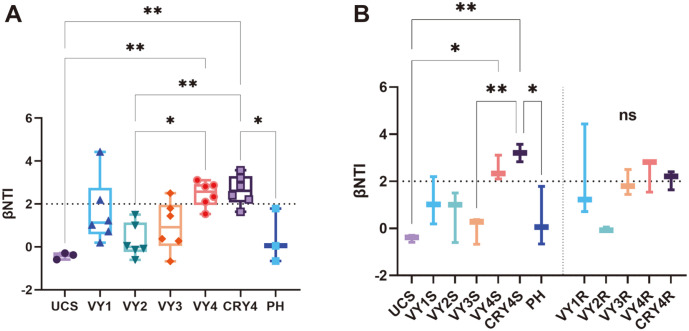
Distribution of β Nearest Taxon Index (βNTI) according to the environmental distance with the indicating assemblage processes of bacterial communities. Assemblage processes of bacterial communities with (**A**) cultivation years and (**B**) different niches. Tukey's multiple comparisons test was performed with various groups to compare the differences. The significance is represented by * (*p* < 0.05) and ** (*p* < 0.01).

The assemblage processes of bacterial communities with American ginseng cultivation years were transformed from stochastic to deterministic processes (Fig. 4A). Compared with natural ecosystems, the long-term cultivation process is more influenced by external conditions, which might lead to phylogenetically non-conserved traits [49]. In the 4^th^ year, the βNTI of the bacterial community significantly differed from a null distribution in the American ginseng cultivation period (median βNTI > 2; Fig. 4A), indicating the dominant role of deterministic processes, according to the framework of Stegen *et al*. [35]. This deterministic process was derived from the combined contribution of soil and ginseng root niches (Fig. 4B).

**Fig. 5:**
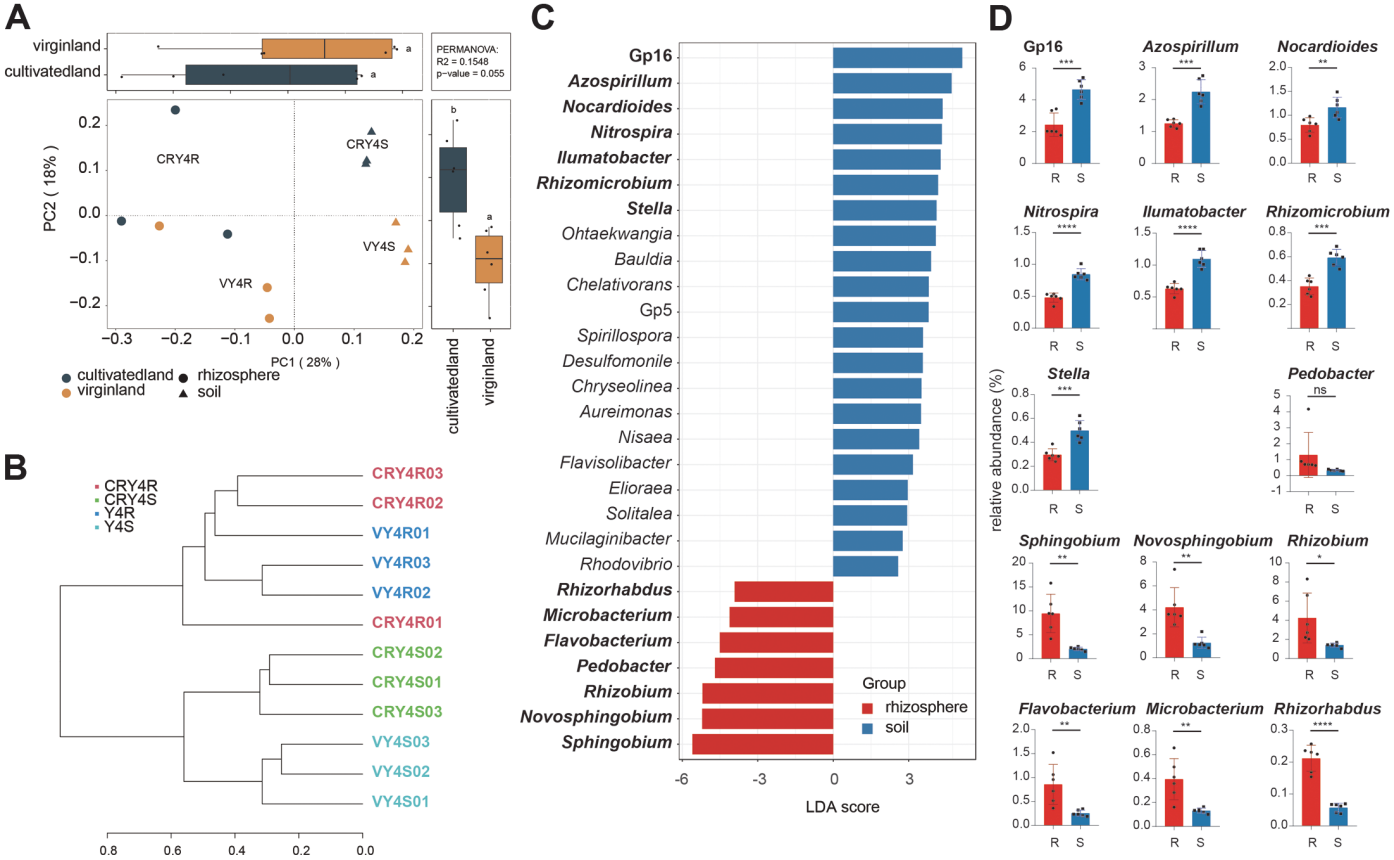
PCoA, Clustering, and LEfSe analyses of ginseng roots and bulk soils in the 4^th^ year of cultivation. (**A**) PCoA, (**B**) Clustering, and (**C**) LEfSe analyses. (**D**) Comparison of relative abundances of the first seven differential genera.

In the current study, we found that different ecological niches had significant effects on the establishment of bacterial communities. The effect of American ginseng on the bacterial community was significantly enhanced in the late cultivation period (Figs. 5A and 5B). Previous studies found that the ginseng root microenvironment can cluster specific microorganisms to degrade toxic compounds and improve disease resistance [50]. Here, American ginseng roots were selective to the composition of the bacterial community. According to the LEfSe analysis, we found that the ginseng roots mainly gathered Proteobacteria of different genera (Figs. 5C and 5D). Previously, *Sphingobium* and *Novosphingobium* had been associated with rhizoremediation [51], biodegradation [52], and plant growth promotion [53, 54]. These genera were clustered and enriched in 4-year-old ginseng roots in our study, indicating that American ginseng roots might promote growth or decompose insoluble matters through symbiosis with *Sphingobium* and *Novosphingobium*. On the other hand, there is a conflicting report in which *Sphingobium* was enriched in root-rot disease soil samples [55], indicating that this genus might also affect the growth of American ginseng as an opportunistic pathogen or a pathogenic factor. Finally, we also observed the enrichment of *Rhizobium* around ginseng roots (Figs. 5C and 5D), which might also contribute to nutrient absorption [56].

### Correlation between Microbial Communities and Soil Nutrients

**Fig. 6:**
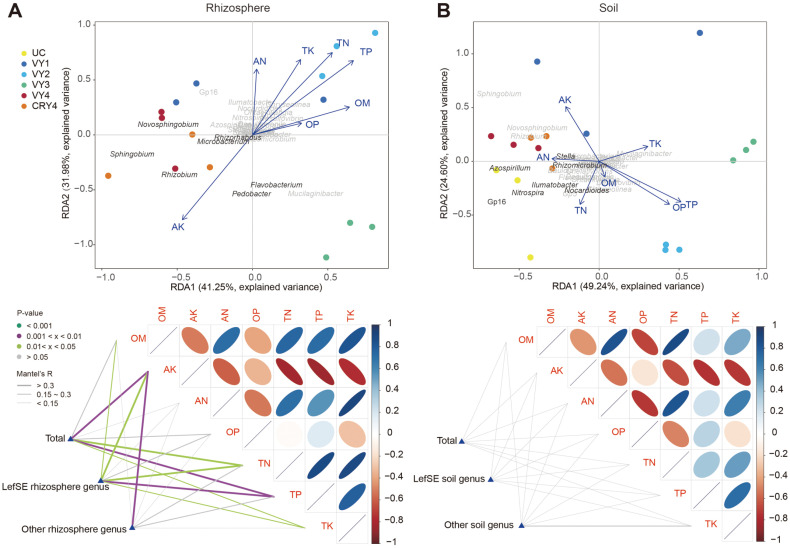
Ordination and relationships between physicochemical variables and the composition of bacterial communities at the genus level after four years of cultivation. RDA and Mantel’s test of (**A**) ginseng and (**B**) soil niches.

**Fig. 7:**
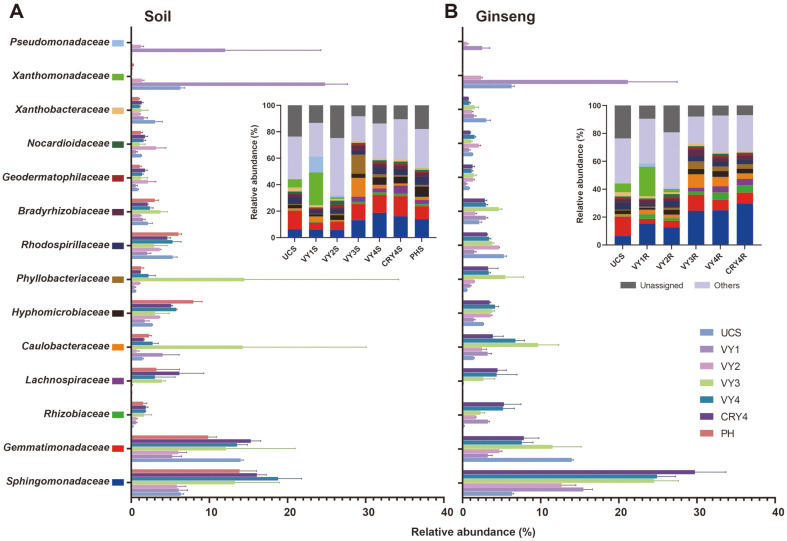
Relative abundances and changes of the top 15 most-abundant bacteria at the family level over American ginseng cultivation years in (**A**) soil and (**B**) ginseng.

Environmental factors can directly or indirectly shape bacterial communities, especially different ecological niches that can shape their specific communities [57]. The root microenvironment, as a means of nutrient exchange and information transfer, affects the accumulation and growth of bacterial communities [58]. In the present study, we found that bacterial communities in different niches had significant effects on soil nutrients (Fig. 6). The AK and TP had the strongest correlation with both discriminative and undifferentiated genera in the ginseng root niche, achieving the highest levels in the fourth year of cultivation (Table 1). Discriminative genera were also strongly correlated with OM, TN, and TK (Fig. 6A). These results suggested that 4-year-old American ginseng roots might select specific bacteria from the soil for uptake of organic matter, nitrogen, phosphorus, and especially potassium. This growth-promoting effect of the rhizosphere microbiome has been confirmed in both field trials and artificial inoculation experiments [59, 60]. Meanwhile, no enrichment of discriminative genera was observed in the first two years of cultivation (Fig. 6A). Therefore, in the future, we will focus on the microbiota enriched in American ginseng roots.

Plants and their microbiotas interact closely with each other, while plants can also promote growth or defense against diseases by shaping the rhizosphere microbiota. The composition of soil microbial communities plays an important role in this process [61, 62]. Interestingly, no significant correlation was found in the American ginseng soil niche in our study (Fig. 6B), in contrast with previous reports [40]. This discrepancy might be attributed to differences in sample processing between studies [20]. According to the soil microbial community assemblage processes (Fig. 4B), we also hypothesized that specific soil bacterial communities might be formed to resist soil-borne diseases or environmental stresses. Further research can focus on the correlation of allelochemicals, environmental factors, and microbiotas.

Overall, we demonstrated that the process of American ginseng cultivation significantly changed the soil chemical properties, bacterial diversity, structure, and composition. Moreover, ginseng roots could selectively enrich root bacterial microbiota from surrounding soils. Compared with uncultivated soils, American ginseng cultivation significantly increased the content of soluble nutrients, resulting in increased soil AN, OP, and AK. The bacterial diversity increased with cultivation years, while 4-year-old ginseng roots affected both the diversity and evenness of bacteria. Proteobacteria, Actinobacteria, Gemmatimonadetes, Acidobacteria, Firmicutes, and Bacteroidetes were the dominant bacterial phyla, regardless of cultivation years and ecological niches. Deterministic processes dominated the bacterial community dynamics in 4-year-old American ginseng cultivation groups for all soil types. Additionally, 4-year-old ginseng roots had a significant effect on the bacterial community. They were enriched in specific genera, including *Sphingobium*, *Novosphingobium*, and *Rhizorhabdus*. These specific genera were mainly associated with variations in AK, TP, TK, and OM. Altogether, these results might help understand the influence of the American ginseng cultivation process and mode in shifting soil chemical properties and bacterial communities, and the effects of bacterial community enrichment in the American ginseng root niche.

## Figures and Tables

**Table 1:** Soil chemical properties for the American ginseng cultivation process.

Cultivation process	OM (g•kg−1)	AK (mg•kg−1)	AN (mg•kg−1)	OP (mg•kg−1)	TN (g•kg−1)	TP (g•kg−1)	TK (g•kg−1)
UCS	23.24 ± 4.88ab	95.03 ± 10.47ab	161.46 ± 8.12ab	17.48 ± 4.4a	2.7 ± 0.42a	0.83 ± 0.19a	10.64 ± 2.37a
VY1	24.12 ± 4.88ab	83.33 ± 19.31ab	129.17 ± 11.75ab	23.79 ± 6.33ab	1.53 ± 0.43a	1.07 ± 0.42ab	17.36 ± 1.18a
VY2	23.24 ± 2.38ab	53.74 ± 9.26a	114.17 ± 6.68a	118.39 ± 10.36ab	1.91 ± 0.42a	1.54 ± 0.11ab	16.68 ± 0.39a
VY3	41.13 ± 1.5b	53.74 ± 19.31ab	114.17 ± 11.75ab	118.39 ± 6.33ab	1.91 ± 0.43a	1.54 ± 0.42ab	16.68 ± 1.18a
VY4	36.78 ± 3.37ab	428.53 ± 43.53b	227.04 ± 1.02b	189.23 ± 16.76b	2.42 ± 0.2a	1.93 ± 0.13b	17.84 ± 1.33a
CRY4	32.61 ± 1.03ab	230.03 ± 4.66ab	198.46 ± 4.79ab	165.51 ± 17.17ab	1.91 ± 0.1a	1.69 ± 0.17ab	17.07 ± 0.19a
PH	20.58 ± 1.99a	68.92 ± 5.33ab	128.29 ± 5.95ab	120.42 ± 2.82ab	1.55 ± 0.1a	1.51 ± 0.06ab	15.88 ± 0.58a

All values are an average from three replicates ± standard deviations. Values with the same letters within a column did not significantly differ based on the Wilcoxon/Kruskal-Wallis test.
